# Modifying Inorganic Structure through Hydration in Vapor Phase Infiltrated AlO_*x*_H_*y*_‑PIM‑1 Hybrid Membranes: Implications for Solvent Stability, Permeance, and Selectivity

**DOI:** 10.1021/acs.chemmater.4c03453

**Published:** 2025-05-27

**Authors:** Benjamin C. Jean, Yi Ren, Ian Slagle, Ryan P. Lively, Faisal M. Alamgir, Mark D. Losego

**Affiliations:** School of Materials Science and Engineering and School of Chemical and Biomolecular Engineering, 1372 Georgia Institute of Technology, Atlanta, Georgia 30332, United States

## Abstract

Vapor-phase infiltration (VPI) presents a promising approach to enhancing the stability of organic membranes in organic solvents while maintaining critical properties, such as membrane permeance and selectivity. However, the precise chemical structure of the infiltrated inorganics and their impact on solvent stability remain poorly understood, limiting efforts to improve VPI treated hybrid membrane technology for organic solvent reverse osmosis (OSRO). This study uses X-ray absorption spectroscopy alongside X-ray photoelectron spectroscopy (XPS) to elucidate the inorganic cluster structure within PIM-1/AlO_*x*_H_*y*_. By analyzing the H_2_O ratio via XPS and assessing the first and second shell Al coordination numbers from Al K-edge extended X-ray absorption fine structure (EXAFS) spectroscopy, we propose that aluminum oxyhydroxide tends to form nonlinear networked structures. X-ray absorption near-edge spectroscopy (XANES) analysis confirms a predominantly six-coordinate structure in the first shell, while EXAFS analysis of the second shell reveals the presence of three aluminum atoms, suggesting clusters significantly larger than simple dimers or trimers, similar to larger aluminum hydroxide and oxyhydroxide crystal structures. Furthermore, we demonstrate that postprocessing techniques, such as dehydration and rehydration, can be utilized to control this network structure and membrane permeance and selectivity without compromising solvent stability.

## Graphical abstract

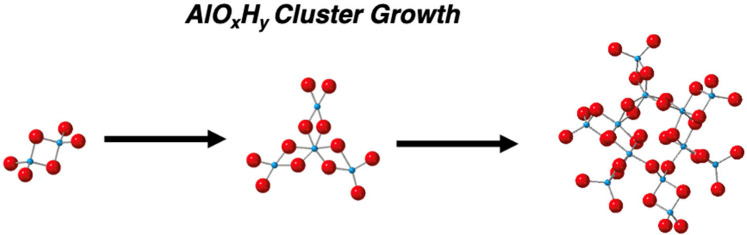

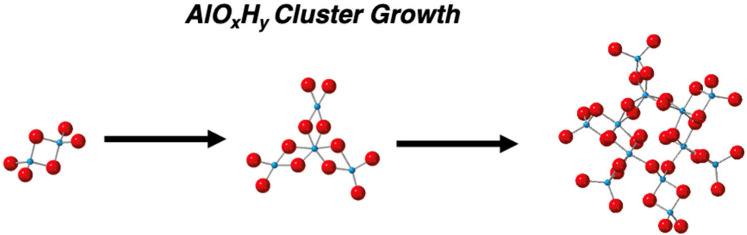

## Introduction

Separation processes to purify chemical substances from mixtures are vital for numerous chemical industries including pharmaceuticals, petrochemicals, and agriculture. However, chemical separations have been estimated to be responsible for as much as 10–15% of global energy consumption.^1^ These high energy demands stem from the current use of energy-intensive, thermally driven separation processes, such as distillation and drying. Membrane technologies that physically and/or chemically separate species can reduce energy use by as much as 90%,^1^ but membrane technologies for separating small organic molecules remain limited in efficiency and long-term reliability. Advances in membrane material chemistry and microstructure are necessary to achieving improvements in chemical separations.

Polymers of intrinsic microporosity, or PIMs, have gained significant attention in recent years due to their exceptional membrane properties. PIMs are characterized by a high degree of microporosity due to their rigid monomer units. The intrinsic microporosity in PIMs leads to excellent membrane permeability and selectivity. As such, PIMs are widely researched and utilized for gas separations, water purification, and even membrane-based catalysis.^2,3^ However, as linear organic polymers, PIMs are still susceptible to swelling and/or dissolution in organic solvents.

Vapor deposition of PIMs has been shown to improve membrane performance in gas phase separations.^2,4^ Recently, we demonstrated that vapor phase infiltration (VPI) of inorganic metal oxyhydroxide clusters significantly improves the chemical stability of PIM-1 membranes in organic solvents, expanding the application space for organic solvent separations. VPI exposes a polymer, like PIM-1, to metalorganic or metal halide vapors that sorb and become entrapped inside the polymer, creating an organic–inorganic hybrid material.^5–7^ VPI has also been used to modify other properties in polymers, including optical properties,^8–10^ electrical conductivity,^11^ thermal stability,^12^ and UV degradation resistance.^13^

In past work, we have connected different physicochemical structural features of the organic, inorganic, and organic–inorganic interactions to the properties of the resultant VPI hybrid material. For example, we have shown that strong primary bonding between infiltrated Al species and the copolymer PS-r-PHEMA forms a network polymer with increased glass transition temperature.^14^ Similar primary bonds form when PMMA is infiltrated with TiCl_4_, but TiCl_4_ appears to act as a multifunctional cross-linker, forming three or four bonds with the polymer, raising the glass transition temperature even more than would be expected for a simple bifunctional cross-linker.^15^ In 2,2′,7,7′-tetrakis-[*N*,*N*-di­(4-methoxyphenyl)­amino]-9,9′-spirobifluorene (spiro-OMeTAD), infiltrated TiO_*x*_ species appear not to chemically bond to the organic conductor but rather act to disrupt the existing π–π stacking, lowering the glass transition temperature but raising the energy barrier for crystallization of the organic phase.^12^ For infiltration of AlO_*x*_H_*y*_ species into PIM-1, the experimental evidence suggests no primary bonding between the organic and inorganic species.^16^ Based on available spectroscopic and computational evidence, we have hypothesized that the AlO_*x*_H_*y*_ species tend to polymerize toward chains or sheets that interpenetrate among the polymer chains, preventing reptation and stabilizing the polymer against swelling and dissolution in good solvents.^17,18^ However, a full understanding of the key structural features in these hybrid materials and how to characterize these structural features to best predict properties is still lacking.

A critical challenge in probing the structure of infiltrated inorganics is their uniform distribution on a molecular scale within an amorphous polymer matrix. While in some cases direct electron microscopy imaging of the structure of these hybrid materials is possible, especially when multiple infiltration cycles are used, often the inorganic species remain amorphous and at the sizes of molecular clusters, precluding clear contrast mechanisms for imaging.^19–21^ Spectroscopies including infrared (IR),^22^ X-ray photoelectron spectroscopy (XPS),^23,24^ and nuclear magnetic resonance (NMR)^18,25^ have proven promising in rationalizing the structure of these hybrids, but most often these techniques are limited to nearest neighbor information. As suggested above, in some cases, it is thought possible to grow the inorganic clusters into chains or other structures, suggesting that more advanced spectroscopic techniques like pair distribution functions (PDF), X-ray absorption near edge spectroscopy (XANES) and extended X-ray absorption fine structure (EXAFS) spectroscopy that generate information about the short-range structure of a material beyond the nearest neighbor could be useful. For example, recently, Hu et al. conducted indium K-edge EXAFS and PDF analysis on InO_*x*_H_*y*_ clusters infiltrated within PS-*b*-PMMA block copolymers, showing potential InO_*x*_H_*y*_ cluster formation pathways.^21^ Additionally, Weisbord et al. showed that zinc K-edge XANES indicates that infiltrated ZnO tends to form tetrahedral complexes.^26^

This article introduces the use of Al K-edge X-ray absorption spectroscopy, encompassing both XANES and EXAFS, to characterize the electronic, chemical, and short-range order structure of AlO_*x*_H_*y*_ species infiltrated into PIM-1. We previously showed that these infiltrated species are susceptible to hydration and dehydration when placed in varying humidity environments.^18^ Here, we use this variation in hydration state as a starting point to probe differences in the structural features of the inorganic and its effects on membrane properties, including chemical solubility, permeance, and separation factor.

## Experimental Methods

### Vapor Phase Infiltration

Vapor phase infiltration of PIM-1 powders, fibers, and thin films was conducted in a custom-built, isothermally controlled hot-wall reactor described previously^27^ and operated with automated sequencing software.^28^ Prior to infiltration, PIM-1 samples were soaked in MeOH for 30 min to reset the polymer free volume and then dried for 1 h in a fume hood. After PIM-1 material was placed in the reactor at 90 °C, the system was purged with N_2_ for 1 h, before 1 h of active pumping. After 1 h of pumping, PIM-1 was exposed to TMA. All samples were infiltrated with TMA at 90 °C for 5 h at 0.3 Torr. After TMA exposure, the system was placed under active vacuum for 5 h before a 5 h exposure to deionized water vapor at 1.8 Torr. After water exposure, the system was held under active vacuum for 5 h before being removed from the reactor.

### Postprocessing

To modify the hybrid membrane’s chemical structure, several postprocessing treatments were considered. Some membranes were dehydrated by heating the hybrid membranes to 120 °C for 90 min in an oven under ambient air conditions. After dehydration, membranes were stored in a desiccant until characterization. A subset of these dehydrated membranes were rehydrated by storing in a humidity chamber at 95% humidity and room temperature for 12 h.

### X-ray Photoelectron Spectroscopy

X-ray photoelectron spectroscopy (XPS) was used to characterize the PIM-1/AlO_*x*_H_*y*_ hybrid powders. XPS was conducted using a Thermo K Alpha XPS instrument with a monochromatic Al Kα X-ray source (1486.6 eV). For all high-resolution scans, 10 scans with a step size of 0.1 eV were taken and averaged over the element’s characteristic emission energy range. All spectra were charge shifted according to the peak position of C–C bonds in the C 1s spectrum, setting its peak position at 284.8 eV.

### X-ray Absorption Spectroscopy

X-ray absorption spectroscopy of PIM-1/AlO_*x*_H_*y*_ hybrids were conducted at the 7-ID tender X-ray beamline at Brookhaven National Lab. All materials were transferred from the synthesis laboratory to the 7-ID beamline in gastight containers containing an inert gas ambient. Powders were mounted onto copper tape at the beamline. Energy was monochromatized at the 7-ID beamline using a 1200 lines/mm grating. We will use the terminology of XANES to describe the near edge portion of the X-ray absorption spectrum, and the term EXAFS to describe the extended portion of the X-ray absorption spectrum. Al K-edge XAS was run from 1360 to 2360 eV, with a step size of 0.3 eV/step between 1540 and 1590 eV near-edge (XANES) region and 0.05 Å^–1^ step sizes between 1590 and 2360 eV in the extended (EXAFS) region. N K-edge measurements were unsuccessful due to the low atomic concentration. Partial electron yield signals were integrated for one second at each energy point. All data processing was done in ATHENA and ARTEMIS XAFS software.

### UV–vis Spectroscopy for Solvent Stability Measurements

UV–vis absorption spectroscopy measurements were used to determine the solvent stability of PIM-1 and hybrid PIM-1 materials in THF. An Avantes Avaspec-2048 UV–vis spectrometer with a halogen light source was used for UV–vis measurements. For all materials, a PIM-1 or hybrid PIM-1 fiber of known mass was placed in a scintillation vial with 20 mL of THF. Because PIM-1 has a distinctive yellow color, its dissolution is readily tracked by measuring the color of the solvent solution. Measurements were taken at regular intervals over the course of several days. Absorption values were translated into concentrations using a set of reference standards and Beer’s Law. Reference standards were prepared by dissolving a known amount of PIM-1 in a known amount of tetrahydrofuran (THF) and collecting the spectra. The calibration curve was used to determine the mass dissolved and percent dissolved.

### Membrane Fabrication and Crossflow

Pristine PIM-1 thin film composite membranes were made via spin coating a PIM-1 topcoat dope onto the porous cross-linked Matrimid support, under 2500 rpm for 5 min with nitrogen flow. The PIM-1 topcoat dope was 1.5 wt % PIM-1 in chloroform that had been sonicated for 1 h and filtered through 0.45 μm PTFE filter. Matrimid support was obtained with a dissolved dope of Matrimid, *N*-methyl-2 pyrrolidone, tetrahydrofuran, ethanol, water, and LiNO_3_ with a mass ratio of 16:69:10:3:1:1. Dissolved Matrimid dope was casted with a 10 mil blade, and after 9 s of solvent evaporation in the hood, Matrimid support was delaminated in DI water. Three times of methanol and hexane solvent exchange was performed, one h in between. Finally the Matrimid support was immersed in cross-linking solution with 5g of xylylene diamine in 100 mL of methanol for 24 h under room temperature. After being washed with MeOH, Matrimid supports were ready for use.

As for membrane crossflow permeation, three thin film membrane cells in series were pressurized by an Azura P 4.1S Knauer pump, with the retentate and permeate recycling back into the feed bottle when not being collected. All membranes were tested under a constant pressure of 40 bar for 5–7 days before the sample was collected for separation performance testing using gas chromatography (Agilent 7890B).

## Results and Discussion

Understanding the physicochemical structure of vapor infiltrated organic–inorganic hybrid materials is critical to informing the design of properties in these materials. Here we use PIM-1 infiltrated with AlO_*x*_H_*y*_ as our system of study and investigate the variations in the inorganic’s structural features upon dehydration and subsequent rehydration of this material for a single process condition (90 °C, 5 h TMA exposure, 1 h purge, 5 h water exposure, 1 h purge, for 1 cycle). As such, we will describe the materials tested as (1) as synthesized hybrid membranes, (2) dehydrated hybrid membranes, and (3) rehydrated hybrid membranes. We first investigate these materials using spectroscopies that probe physicochemical information limited to mostly the first coordination shell, then with EXAFS spectroscopy to probe more exterior coordination shells, and finally the separation performance of the membranes is measured and correlated to the differences in structural features that have been determined.

### Structural Characterization within the First Coordination Shell

XPS spectra of PIM-1 and PIM-1/AlO_*x*_H_*y*_ are collected to characterize the first coordination shell binding environments in PIM-1/AlO_*x*_H_*y*_ hybrids. Figure 1a shows the XPS spectra of the O 1s for the as-synthesized PIM-1/AlO_*x*_H_*y*_ hybrid. Consistent with previous findings, the O 1s XPS indicates 3 peaks. The highest energy peak at 533.5 eV is also seen in pure PIM-1 and is a result of the C–O bonds in the polymer.^18^ This peak could also overlap with H_2_O, which has a binding energy at ∼533.4 eV, which we will return to later.^29^ The other two peaks are Al–OH at 531.5 eV and Al–O at 530.4 eV.^18,29^

**Figure 1:**
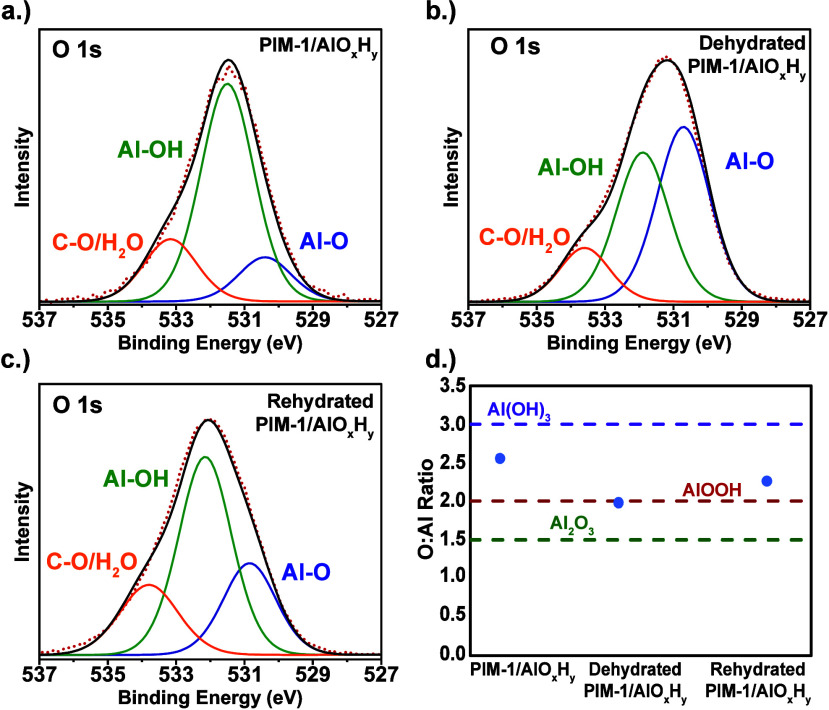
High-resolution XPS O 1s spectra for (a) as synthesized PIM-1/AlO_*x*_H_*y*_ hybrid membranes, (b) dehydrated PIM-1/AlO_*x*_H_*y*_ hybrid membranes, and (c) rehydrated PIM-1/AlO_*x*_H_*y*_ hybrid membranes. (d) O/Al ratio for each of these hybrid membranes based on XPS survey spectra.

Figure 1d plots the relative ratios of O:Al as measured from the XPS survey scans; this ratio is indicative of various hydrated states of alumina, for example, Al_2_O_3_ versus AlOOH versus Al­(OH)_3_, which have O:Al ratios of 3:2, 2:1, and 3:1, respectively. Note that to get an accurate O:Al ratio, only oxygen from the Al–O and Al–OH peaks is included, while O from the C–O/H_2_O peak is excluded; a similar approach was taken by Liu et al.^18^ Interestingly, the as-synthesized PIM-1/AlO_*x*_H_*y*_ hybrid shows a significant amount of Al–OH with an O:Al ratio approaching the gibbsite (Al­(OH)_3_) phase. Upon dehydration (Figure 1b,d), the relative amount of hydroxide decreases, while the oxide fraction (Al–O) increases, indicating potential condensation reactions of the hydroxyl groups. The O:Al ratio in this material matches well to the boehmite (AlOOH) phase. Upon rehydrating this dehydrated membrane (Figure 1c), the oxide fraction again reduces and more hydroxide returns, although not as much as the as-synthesized material. This result is expected given that the formation of Al­(OH)_3_ is thermodynamically favored for alumina at these temperatures and high humidity.^30^ Interestingly, only some of the oxide reverts back after rehydration, leaving approximately twice the amount of oxide in the rehydrated material compared to the as-synthesized PIM-1/AlO_*x*_H_*y*_. Concurrently, the C–O/H_2_O peak also increased upon rehydration. This increase suggests that some water ligands (H_2_O) are coordinating to Al rather than converting the Al–O bonds back to Al–OH bonds during rehydration.

We have previously proposed that water molecules may coordinate to Al to help fill out the octahedral aluminum coordination sphere and the amount of water might be controlled by postprocessing conditions.^18^ To further investigate this claim, we attempt to evaluate the amount of water detected in these XPS spectra by calculating a H_2_O:Al ratio. Recall that the C–O and H_2_O binding energies overlap and cannot be separated spectroscopically. Instead, we determine this water content by comparing measured results to stoichiometric expectations. Briefly, we do this by first calculating the atomic percent of O in just the organic portion of the material (the 533.5 eV peak, which overlaps C–O and H_2_O) by multiplying the fraction of O in the organic determined from the high-resolution 1s spectrum by the total O content determined from the XPS survey scan. We then determine the stoichiometrically expected amount of O in the material based on multiplying the atomic fraction of O expected in the PIM-1 monomer by the total amount of O measured in the XPS survey spectrum. In all cases, we find this stoichiometrically expected amount to be lower than the measured amount, and we attribute the excess to O in water ligands bound to the aluminum. A more detailed exemplary calculation is included in SI.

Figure 2 plots the calculated H_2_O:Al ratio for each hybrid membrane. Upon dehydration, the water content decreases from 0.14 to 0.09. However, after rehydrating, the water content increases significantly to nearly 0.35. Likely, on exposure to humidity, some waters rehydrate oxide bonds into hydroxide bonds, while other waters coordinate to aluminum to fill the coordination sphere. Interestingly, though, the amount of coordinated water estimated here is significantly less than that proposed in our prior work, which used simulations to predict the structure of simple aluminum oxyhydroxide dimers. These previously proposed dimers have an H_2_O:Al ratio of 2:1 (2.0). However, here the H_2_O:Al ratio is at most about 1:3 (0.33) in the rehydrated membranes but below 1:7 (0.14) in the other hybrids. This result suggests that the structure of the inorganic in these hybrids is likely much more complex than a simple dimer. We discuss how this lower than previously expected water ligand content supports the proposition of a new structure for the inorganic constituents in more detail below.

**Figure 2:**
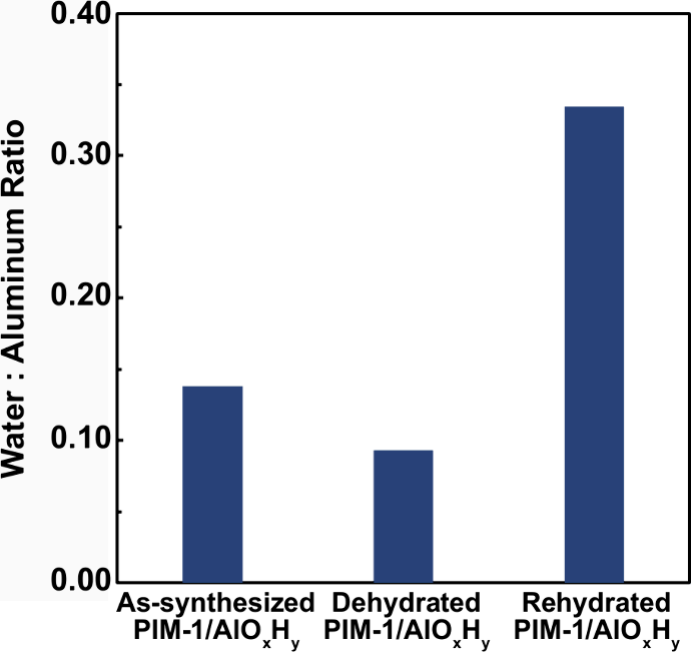
Plot of the H_2_O:Al ratio determined from XPS in the PIM-1/AlO_*x*_H_*y*_ hybrid for the as-synthesized, dehydrated, and rehydrated conditions.

Figure 3 presents the Al K-edge XANES spectra collected for the as synthesized PIM-1/AlO_*x*_H_*y*_ hybrid membranes, again at varying degrees of hydration. Al K-edge XANES spectra can detect variations in the Al coordination sphere and are particularly sensitive to the coordination number.^31,32^ Tetrahedrally coordinated aluminum, ^[4]^Al, has a lower energy electronic transition than octahedrally coordinated aluminum, ^[6]^Al, due to the symmetry of the orbital mixing, resulting in a lower energy absorption edge. ^[6]^Al also commonly exhibits a doublet spaced by ∼4 eV.^31,33^ Distortions or chemical differences in octahedral coordination spheres can cause variations in the peak intensity ratio of this doublet.^32,34^ Figure 3a shows the offset XANES spectra for all three hybrid membranes (as synthesized, dehydrated, and rehydrated). Notably, each spectrum shows multiple peaks with varying peak intensities, indicating that dehydration and rehydration cause inorganic rearrangements that alter the inorganic structure.

**Figure 3:**
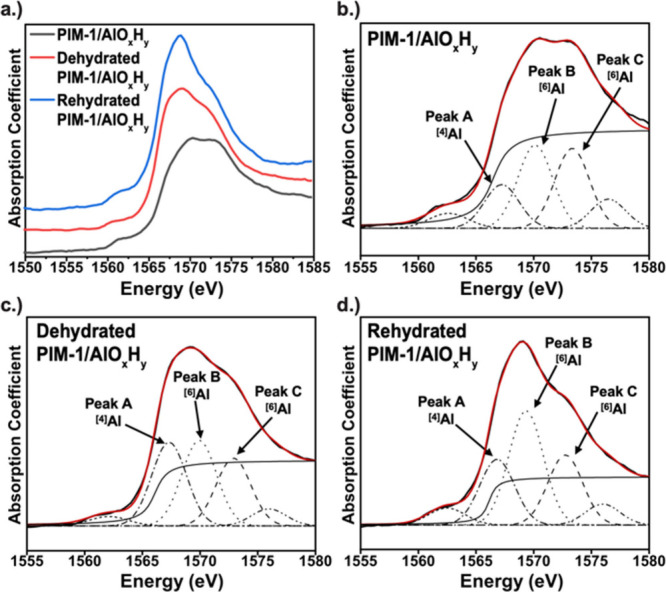
(a) XANES spectra for all processing conditions; (b) fitted XANES spectrum for PIM-1/AlO_*x*_H_*y*_; (c) fitted XANES spectrum for dehydrated PIM-1/AlO_*x*_H_*y*_; and (d) fitted XANES spectrum for rehydrated PIM-1/AlO_*x*_H_*y*_.

To gain more direct insight into these structural changes, an in-depth analysis of the XANES spectra, including peak fitting, is undertaken. A fwhm of 1.5 eV is used for all peaks, consistent with values used for other Al K-edge XANES fittings.^36,37^ Figure 3b–d shows the deconvoluted XANES spectra for the various hybrid PIM-1/AlO_*x*_ materials. Here, for simplicity, the major peaks are labeled Peak A, B, and C. Major peaks at 1569.1 eV (B) and 1572.3 eV (C) are observed, consistent with octahedral ^[6]^Al, and a shoulder peak at 1566.2 eV (A) is indicative of tetrahedral ^[4]^Al. Lastly, the unlabeled peak at ∼1576 eV is from the first EXAFS oscillation beyond the XANES region. Table 1 summarizes these peak locations and compares them to those reported for pure corundum and gibbsite.^35^ Note that while gibbsite and corundum should both contain only ^[6]^Al, measurements of real materials often exhibit detectable amounts of ^[4]^Al, as reported in this table.^38,39^ Interestingly, each hybrid material studied here also shows a pre-edge feature at ∼1563 eV. Computational Al K-edge XANES simulations have suggested that such pre-edge features are consistent with the formation of inorganic aluminum oxyhydroxide clusters of several or more aluminum atoms,^40^ providing some evidence that the inorganics in these hybrids are possibly networking with each other.

**Table 1:** Summary of Al K-Edge XANES Peak Positions for Each Processing Condition Compared with Reference Values for Gibbsite and Corundum^35^

	Peak A (eV)	Peak B (eV)	Peak C (eV)	B/C ratio
As-synthesized PIM-1/AlOx	1566.2	1569.1	1572.3	0.94
Dehydrated PIM-1/AlOx	1566.8	1569.3	1572.4	0.79
Rehydrated PIM-1/AlOx	1566.3	1568.8	1572.2	0.61
Gibbsite	1566	1568.5	1571	1
Corundum	1566	1568.7	1572.2, 1571	0.51

Comparing Figures 3b, 3c, and 3d, we observe a continuous change in the XANES spectrum upon dehydration and rehydration of the hybrid membranes, suggesting changes in coordination. Notably, as indicated in Table 1, the positions of the A, B, and C peaks remain constant upon dehydration and rehydration, but the relative intensities fluctuate. Changes in intensities of the A and B peaks are typically interpreted as indicative of changes in the coordination state from ^[4]^Al to ^[6]^Al. The integrated intensity ratio of these two peaks can be used to estimate the relative amounts of ^[6]^Al and ^[4]^Al.^37,38^ Using this method, we calculate 67% ^[6]^Al in the as-infiltrated material, 51% ^[6]^Al in the dehydrated material, and 62% ^[6]^Al in the rehydrated material. These values are slightly lower than those previously measured by ^27^Al NMR (76%),^18^ but prior reports have also suggested that these XANES calculations tend to overpredict the amount of ^[4]^Al compared with NMR studies.^33^ Notably, the coordination number as measured by XANES under vacuum conditions is similar to the coordination numbers determined via NMR under ambient conditions. This indicates that these clusters are fairly stable under vacuum as well as ambient conditions.

While rehydration appears to nearly restore the relative octahedral to tetrahedral ratio, ^[6]^Al: ^[4]^Al, clear differences exist in the XANES spectra for the as-synthesized (Figure 3b) and rehydrated (Figure 3d) materials. Specifically, the B and C peaks have different relative intensities, as indicated in Table 1. Changes in the B and C peak intensities are commonly indicative of distortions and/or bond length changes in the Al octahedral coordination environment.^36^ Interestingly, the B/C ratio for as-synthesized PIM-1/AlO_*x*_H_*y*_ (0.96) closely resembles that reported for gibbsite (1.0).^36^ However, after dehydration, the B/C ratio decreases, and this decrease continues upon rehydration, indicating changes in the Al coordination sphere beyond changes in the coordination number.

### Consequences of First-Shell Coordination on Proposed Structure

In a prior publication, we report on the possibility of these PIM-1/AlO_*x*_H_*y*_ materials containing aluminum oxyhydroxide dimers or possibly trimers.^18^ In this prior report, first-principles calculations indicated that bridging hydroxyls are more energetically favorable to form than bridging oxide bonds. Moreover, the 1- oxidation state of hydroxyls compared to the 2- oxidation state of oxide bridges makes higher coordinations for the 3+ Al atoms more achievable. We thus suggested that “linear” aluminum oxyhydroxide structures, as depicted in Figure 4 *structure (a)*, were forming. For *structure (a)*, the ^[6]^Al coordination is achieved through uncharged water ligands or possibly coordinations to nitrile groups on the PIM-1 chain. However, the new data presented herein contradict some features in this structure. Most prominently, the H_2_O:Al ratio determined from XPS and reported in Figure 2 is always below 0.35 and near 0.10 for the as-synthesized hybrid material. These values are significantly below the ratio of 2.0 expected for the dimer. Moreover, even if these linear aluminum hydroxide chains were infinitely long, the expected ratio of H_2_O:Al would still be 1.0. Thus, these data suggest that these clusters are likely forming additional hydroxide bridges and not being satisfied by water ligands to achieve the predominant 6-fold coordination observed in XANES and NMR.

**Figure 4:**
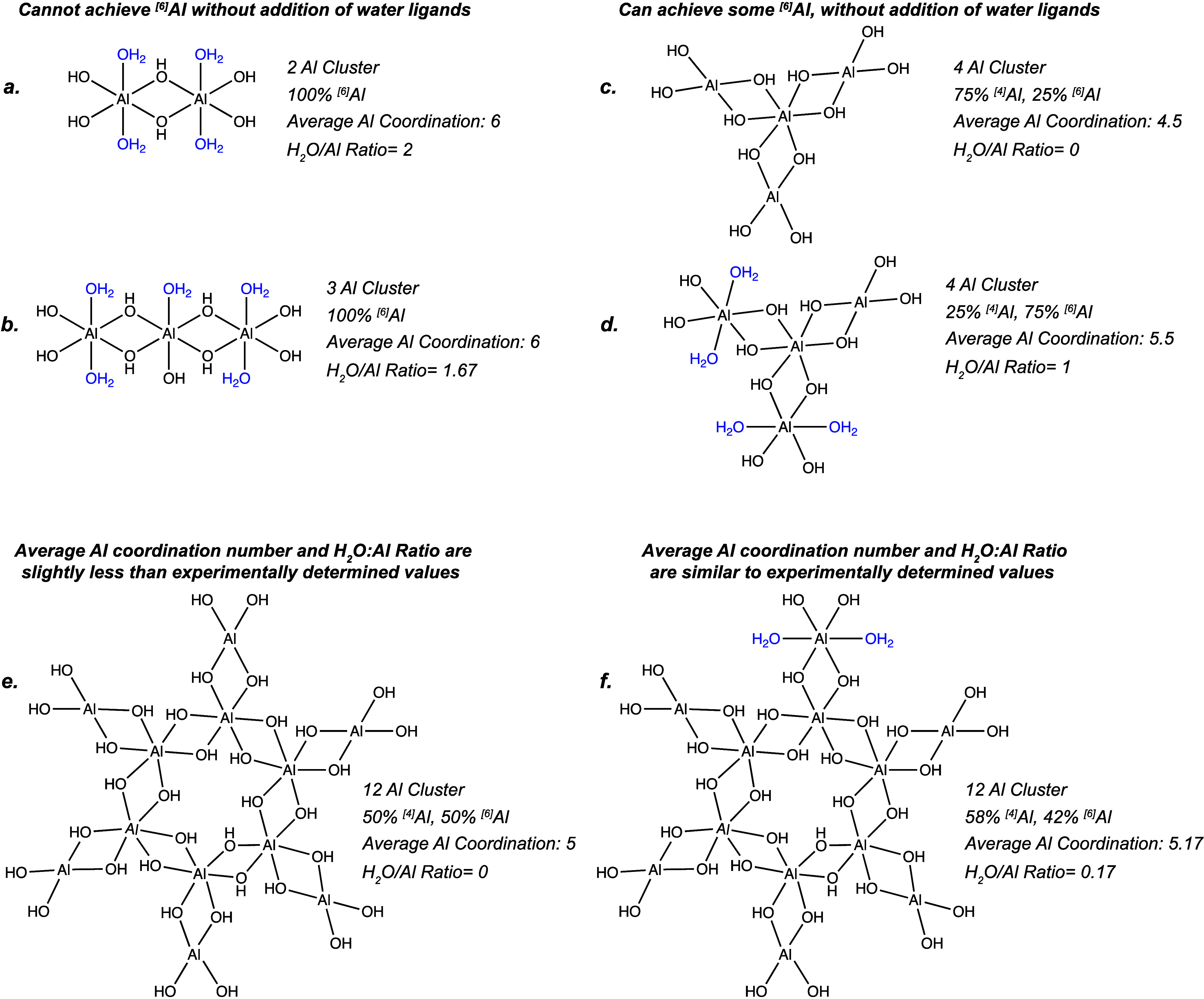
Comparison of cluster size, average coordination number, and H_2_O:Al ratio for several possible Al­(OH)_3_ clusters ranging from linear chains to more networked structures.

Figure 4 shows some alternative aluminum hydroxide clusters that have been proposed to form during gibbsite nucleation and growth.^41,42^ These structures demonstrate how nonlinear networking in these clusters can achieve largely octahedral ^[6]^Al coordination with minimal water ligands present while the presence of edge and end groups provide sites for tetrahedral ^[4]^Al coordination or water ligand binding which are detected but in minority quantities. As shown, generating primarily octahedral aluminum structures with minimal water ligands requires the formation of sufficiently large aluminum clusters. Spectroscopic evidence points to a hydroxide-like network structure that is predominantly octahedral with a small fraction of water ligands present. *Structures (e)* and *(f)* depict networked configurations, both exhibiting at least 50% octahedral coordination. Among these, *Structure (f)* aligns more closely with current spectroscopic data, providing a better representation of the material’s structure compared to earlier proposed linear models. In the next section, we present more direct evidence from EXAFS.

### Structural Characterization Beyond the First Coordination Shell

To provide further evidence of networked clusters forming in these hybrid materials, we carried out extended X-ray absorption fine structure (EXAFS) spectroscopy measurements at the Al K-edge to gain radial distribution information around the aluminum atoms. These data are useful in probing the short and medium range order of these inorganic structures beyond the first coordination shell. Figure 5 plots the EXAFS spectra for the as-synthesized PIM-1/AlO_*x*_H_*y*_ hybrid materials. In the EXAFS spectrum, three distinct peaks are observed; the first two peaks correspond to the characteristic bond lengths of Al–O and Al–Al bonds in gibbsite and corundum. As such, we attribute these peaks to single scattering actions between Al–O and Al–Al. This suggests that even for one cycle of infiltration, infiltrated aluminum tends to form clusters that are at least dimerized, leading to observable Al–Al scattering interactions in the EXAFS spectrum. This provides the first direct evidence of AlO_*x*_H_*y*_ cluster formation during infiltration.

**Figure 5:**
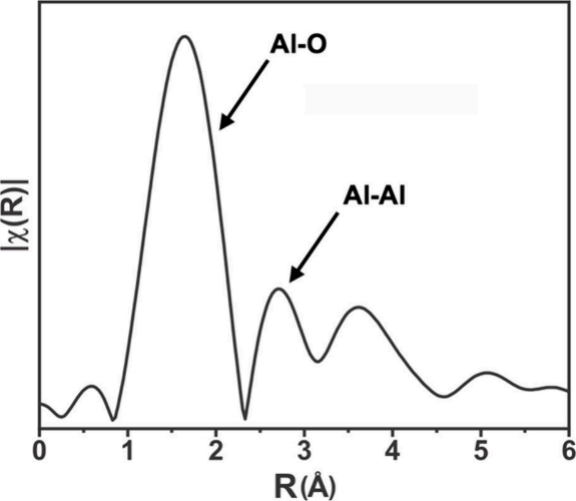
Al K-edge EXAFS spectrum of as-synthesized PIM-1/AlO_*x*_H_*y*_.

EXAFS fitting can provide further information about the aluminum coordination environment beyond the first coordination shell. Figure 6 plots the EXAFS and EXAFS model fits for as-synthesized, dehydrated, and rehydrated PIM-1/AlO_*x*_H_*y*_. Here, EXAFS fitting was performed by using the ARTEMIS software. To fit the EXAFS spectrum, an Al­(OH)_3_ cluster was used as a model molecule. Only Al–O and Al–Al single scattering interactions in the first two coordination shells were included in the fit, and thus, a cluster size larger than 4 Al atoms (*structure (c)*) was not needed for the fit. The fit was not extended beyond the second coordination shell (Al–Al distance) because several multiple scattering (MS) interactions become more pronounced, complicating the fit beyond 3.1 Å. Using these known distances, only minor structural refinement was needed to achieve a good fit to the experimental data.

**Figure 6:**
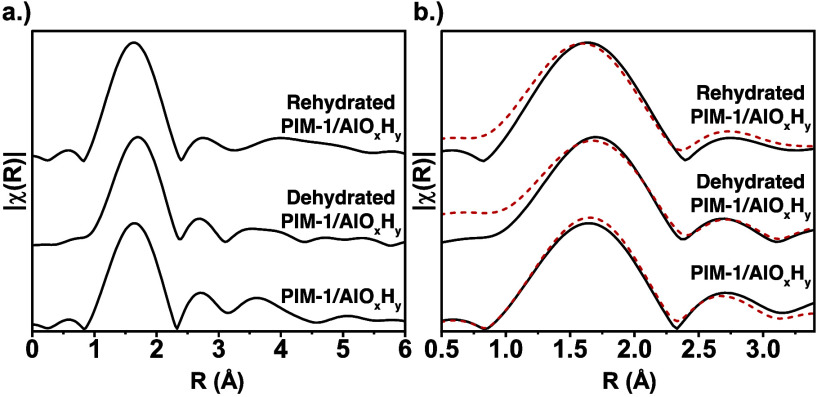
(a) As measured, i.e., without phase shift correction Al k-edge EXAFS spectra for as-synthesized, dehydrated, and rehydrated PIM-1/AlO_*x*_H_*y*_ hybrids. (b) Same spectra showing a fit (dotted red lines) obtained using the ARTEMIS software and an Al­(OH)_3_ cluster as depicted in Figure 4.

Table 2 outlines the bond length, coordination number, and σ^2^ values determined from the EXAFS fitting in ARTEMIS. During EXAFS fitting, the first shell (1 to 2.3 Å) is fit before extending the fitting range to include both the first and second shell (1 to 3.1 Å). For each EXAFS spectrum, the Al–O degeneracy (coordination number) is constrained to values determined from the XANES peak fitting and used to determine the amplitude reduction factor, *S*_0_^2^. This *S*_0_^2^ value is then kept constant for each scattering interaction. For this work, *S*_0_^2^ for PIM-1/AlO_*x*_H_*y*_ was found to be 0.89 (as-synthesized), 0.87 (dehydrated), and 0.92 (rehydrated). The Debye–Waller effect is constrained to values common for aluminum structures (0.001 to 0.003 Å^2^).^43,44^ From these constraints, we are able to determine the bond lengths as a fitting parameter. The Al–O bond length is found to be about 1.9 Å, consistent with known values for aluminum hydroxide.^43,45^ Similarly, the Al–Al distance is determined to be almost 3.0 Å, consistent with prior reports.^43^ Also as expected, the Al–O degeneracy (first shell coordination number) changes with the hydration conditions. However, the Al–Al degeneracy in the second coordination shell remains nearly constant at about 2.7 irrespective of the hydration state. This coordination value is similar to what has been reported for amorphous aluminum hydroxide.^43^ A perfect gibbsite structure would have an Al–Al degeneracy of 3 (3 aluminum atoms in the second shell), while a linear aluminum hydroxide chain would have an Al–Al degeneracy of only 2. Thus, this second shell coordination of 2.7 is indicative of a networking structure as depicted in Figure 4 *structures (e) and (f)*. The consistency in the degeneracy of this scattering path with hydration state further indicates that while postprocessing conditions induce changes to the first shell, larger changes to the general cluster structure are not occurring. For example, if the inorganic clusters were breaking up into smaller clusters, the degeneracy of the Al–Al scattering path would decrease. Because no significant changes are observed in the Al–Al scattering path, this is likely not occurring. The larger structure of these clusters is likely set during synthesis, and dehydration/rehydration induces only smaller chemical changes to the Al–O bonding chemistry (e.g., hydroxide bridges versus oxide bridges vs water ligands).

**Table 2:** Summary of EXAFS Fitting Parameters

Sample	Single Scattering Path	Coordination Number	R (Å)	σ^2^ (Å^2^)
PIM-1/AlO_*x*_H_*y*_	Al–O	5.4	1.94	0.001
	Al–Al	2.7	2.96	0.003
Dehydrated PIM-1/AlO_*x*_H_*y*_	Al–O	5.0	1.99	0.001
	Al–Al	2.7	2.95	0.003
Rehydrated PIM-1/AlO_*x*_H_*y*_	Al–O	5.4	1.95	0.001
	Al–Al	2.7	2.98	0.003

Author: In addition to inorganic cluster size, XPS and XANES have indicated changes in the coordination sphere such as changes to the percent hydroxide and fraction of water ligands present. Figure 7 shows a proposed AlO_*x*_H_*y*_ cluster structure under varying degrees of hydration based on all of these observations. Here, the Al­(OH)_3_ cluster, which is consistent with the structure of small molecules formed during gibbsite nucleation and growth,^42^ consists of both ^[4]^Al and ^[6]^Al. As shown, most of the inorganic structure is set in the as-synthesized structure (*structure (g)*), with only minimal changes occurring upon dehydration (*structure (h)*) and rehydration (*structure (i)*). The as- synthesized structure starts as a primarily ^[6]^Al gibbsite-like structure. Consistent with XPS and XANES, after dehydration, some of the hydroxide ligands are converted to oxides, resulting in a lower average Al coordination number for the structure. After rehydration, some but not all of these oxide ligands are converted back to hydroxide ligands, while additional water molecules coordinate to the Al to fill out the coordination sphere resulting in a primarily octahedral Al structure with a slightly different coordination environment than the initial as-synthesized structure.

**Figure 7:**
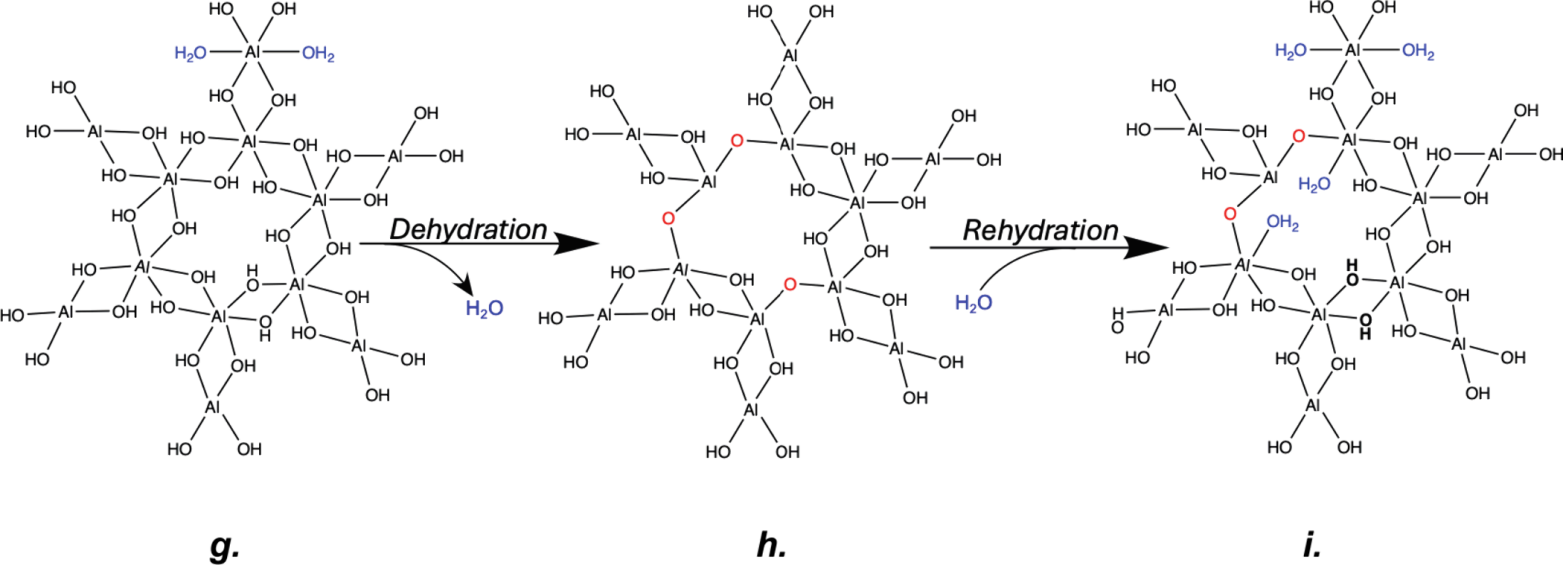
Proposed evolution of AlO_*x*_H_*y*_ clusters within the PIM-1/AlO_*x*_H_*y*_ hybrid materials upon dehydration and rehydration.

The importance of the proposed structures in Figure 7 is that many of the aluminum atoms form six hydroxide bridges to three different next-nearest-neighbor aluminum atoms in the second shell. This leads to a ring structure with diameters of between 5.5 Å (as measured between Al atoms) and 6.4 Å (as measured between bridging hydroxyls), which approach the size of the pores in PIM-1 (∼7 Å).^46^ Thus, it may be possible for such an inorganic cluster to form inside the PIM-1 porosity and serve to “prop” open the pore structure in the hybrid material. Furthermore, we expect the aluminums on the exterior of these rings have the possibility to further network and connect to neighboring 6-member aluminum hydroxide rings; that is to say, what is shown here is a fundamental building block for the inorganic network that is forming, but it has the potential to network to an even greater extent within the hybrid material.

### Degree of Hydration Effects on Membrane Performance

Previously, our group has reported on the applications of PIM-1/AlO_*x*_H_*y*_ membranes in organic solvent reverse osmosis (OSRO).^17^ Here, we further explore the solvent stability and OSRO performance under varying postprocessing conditions in order to clarify how degree of hydration and changes to the inorganic structure modify the membrane performance. Figure 8(a) shows the solvent stability of PIM-1/AlO_*x*_H_*y*_ at 10 wt % inorganic loading in THF under various postprocessing conditions. Each hybrid performs similarly, having an order of magnitude greater stability than the pure PIM-1 polymer. Thus, postprocessing hydration/dehydration appears to have little effect on the membrane solvent stability. This consistency is likely because, as posited earlier, the inorganic cluster structure and size are set in the as-synthesized PIM-1/AlO_*x*_H_*y*_ material. Dehydration and rehydration primarily modify the first shell coordination number and bonds present, not overall networking nor cluster size.

**Figure 8:**
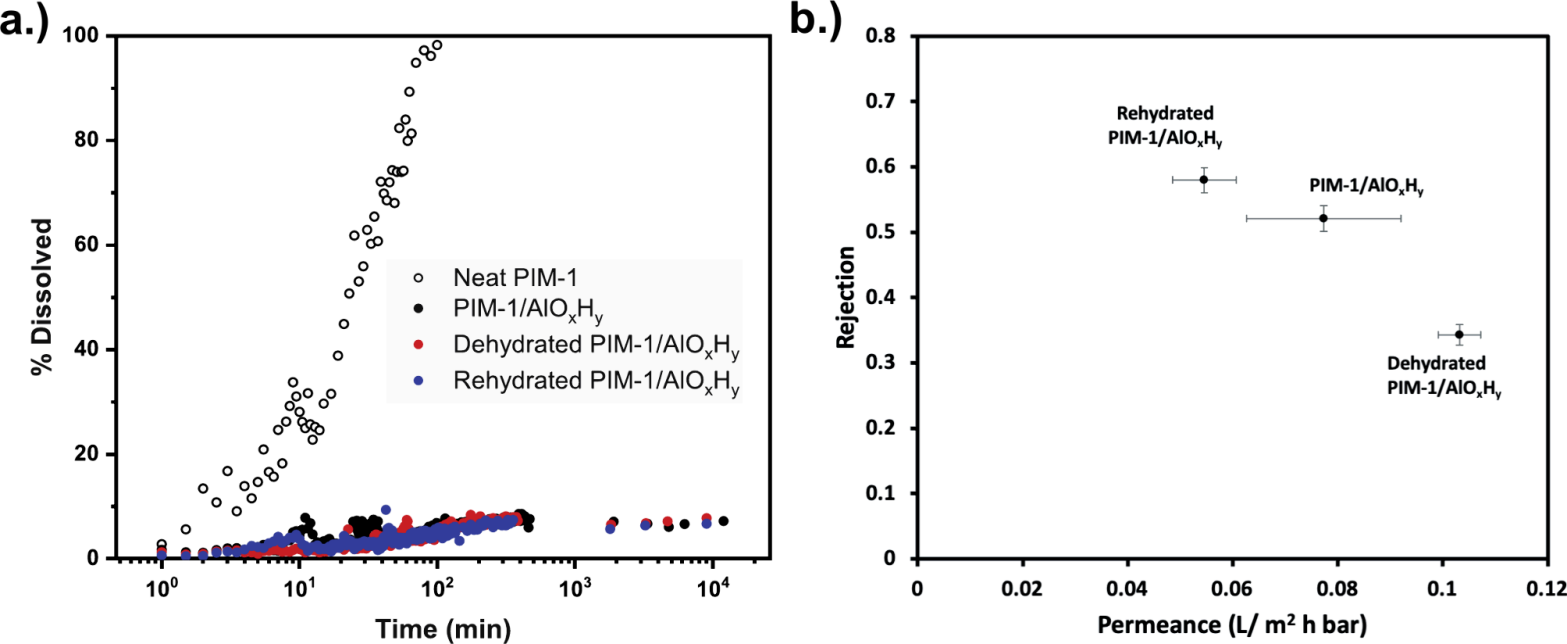
(a) PIM-1/AlOx hybrid membrane solvent stability as compared to pure PIM-1 for the as-synthesized, dehydrated, and rehydrated materials. (b) Plot of permeance vs rejection for each of the hybrid PIM-1/AlO_*x*_H_*y*_ membranes for an OSRO separation of an (90 mol % ethanol/10 mol % iso-octane) mixture.

Figure 8(b) plots the PIM-1/AlO_*x*_H_*y*_ membrane permeance versus rejection for ethanol/iso-octane separations. Rejection is defined by 1 – *C*_*permeate*_/*C*_*feed*_, where a rejection of 100% indicates a total removal of iso-octane in the permeate and a rejection of 0% means that no separation is occurring since permeate and feed are identical. Similar to most membranes, a decrease in permeance is usually accompanied by an increase in the rejection (increased separation selectivity). Here, we observe the most significant change upon dehydration, which leads to higher permeance and less rejection. Upon dehydration, coordination of aluminum’s first shell is decreased. As pictured in Figure 7, this reduced coordination will generate larger porosity in the structure, potentially leading to higher permeance and less rejection. Upon rehydration, membrane properties return to near those of the originally infiltrated material. This result is consistent with the structure returning to similar (higher) first-shell coordination environments upon rehydration. Interestingly, a small difference is observed between the as-infiltrated and rehydrated materials; these differences may be indicative of the believed increase in water ligand coordination occurring in the rehydrated membranes which potentially “repels” hydrophobic iso-octane leading to a slight enhancement in rejection. These ligands are likely larger in size but also potentially more mobile.

These findings suggest that the structural and compositional changes induced by postprocessing primarily impact the transport properties of the membrane without significantly altering its solvent stability. Thus, postprocessing can be used to fine-tune permeance and selectivity without compromising the membrane’s resistance to dissolution. In our prior publication, we demonstrated how these AlO_*x*_H_*y*_-PIM-1 hybrid membranes exceeded the separation performance for ethanol/iso-octane mixtures compared to all prior technologies.^17^ In this current work, our ability to more finely control the chemical structure of these hybrid membranes through dehydration–rehydration has led to a 10 to 15% further increase in separation performance. Thus, more sophisticated understandings of the chemical structure in these organic–inorganic hybrid materials, beyond just the amount of inorganic loading, are likely needed to truly achieve intentional design of their properties.

## Conclusions

This work provides insight into the physicochemical structure of inorganic clusters formed inside of PIM-1/AlO_*x*_H_*y*_ hybrid membrane materials created via vapor phase infiltration. XANES confirms that the aluminum species in these clusters are predominantly 6-fold coordinated with a minority fraction of 4-fold coordinated aluminum. XPS analysis reveals that these inorganic clusters have fewer water ligands than would be expected for linear AlO_*x*_H_*y*_ clusters with predominantly 6-fold coordinated aluminum, suggesting a more networked structure. This proposed networking is further substantiated by EXAFS data that reveals that aluminum’s second coordination shell on average contains 2.7 aluminum atoms, approaching the 3-fold coordinated structure expected for a gibbsite-like network. This work also demonstrates that dehydration and rehydration can alter this physicochemical structure, but predominantly in just the first coordination shell, with the ability to somewhat reversibly transition between hydroxide and oxide linkages and to add or remove waters ligands coordinated to the aluminum centers. However, these variations in hydration do not appear to significantly alter the overall cluster structure, leading to no noticeable changes in the hybrid membrane’s chemical stability in organic solvents, but, due to the first shell chemical changes, differences in membrane permeance and selectivity do occur. Overall, this study demonstrates the complexity of these hybrid materials and the metal oxyhydroxide clusters and how advanced spectroscopies that probe both nearest neighbors and farther coordination shells can be useful in unraveling the physicochemical structure of these hybrids and then connecting these structural changes to observed properties.

## Supplementary Material



## Notes

Determination of water content in PIM-1/AlO_*x*_H_*y*_, and additional informationa bout XANES and EXAFS (PDF)
