# Supplementary material for: Modifying Inorganic Structure through Hydration in Vapor Phase Infiltrated AlO x H y ‑PIM‑1 Hybrid Membranes: Implications for Solvent Stability, Permeance, and Selectivity
Source: Chem Mater. 2025 May 27;37(11):3959–68. doi: 10.1021/acs.chemmater.4c03453 (PMC12225022; doi:10.1021/acs.chemmater.4c03453)
Supplement: Supplementary file 1 [file cm4c03453_si_001.pdf]

Supporting Information: Modifying Inorganic Structure through Hydration in Vapor Phase Infiltrated AlOxHy-PIM-1 Hybrid Membranes: Implications for Solvent Stability, Permeance, and Selectivity

Benjamin C. Jean, † **Yi Ren,** ‡ **Ian Slagle,** † **Ryan P. Lively,** ‡ **Faisal M. Alamgir,** † **and Mark D. Losego** *,†

†School of Materials Science and Engineering, ‡School of Chemical and Biomolecular Engineering, Georgia Institute of Technology, Atlanta, GA 30332, United States

Determining water content in PIM-1/AlO$_x$H$_y$

Below is exemplary data collected from the as-synthesized PIM-1/AlO_xH_y hybrid material. This data is used to provide a sample calculation for determining the H_2O:Al ratio in this material.

XPS Survey spectrum:

C: 45.4 at. %
O: 41.3 at. %, which splits into:
 8 at. % O in polymer based on O 1s spectrum (see below)
 33.3 at. % O due in inorganic based on O 1 spectrum (see below)
Al: 13 at. %

O 1s spectrum:

Al-O: 13.4%
Al-OH: 67.1%
C-O/H_2O: 19.5%

Oxygen attributed to Polymer/ H_2O: 19.5%
Oxygen attributed to inorganic: 80.5%

Determining the Amount of O in the Organic Portion of the Hybrid from XPS Spectra

From the XPS survey spectrum, we know that this material has 41.3 at% O. From the O 1s spectrum, we know that 19.5% of this oxygen is either in the polymer or water and 80.5% of this oxygen (13.4% + 67.1%) is in the inorganic. Multiply the fractions from the O 1s spectrum by the amount of total O in the survey spectrum, we can determine the atomic percents of oxygen in the organic and inorganic components. Thus, the amount of oxygen in the organic components (PIM-1 or water) is 19.5%*41.3 at. % O = 8 at.% O and the amount of oxygen in the inorganic components is 80.5%*41.3 at% O = 33.3 at% O.

Thus, the entire material contains 8 at % O in the organic components based on the above analysis.

Determining the Stoichiometrically Expected Amount of O if Organic Fraction is only PIM-1:

The expected O:C ratio for PIM-1 can be determined from the stoichiometry of its monomer chemistry. A PIM-1 monomer repeat unit has 4 oxygen atoms and 29 carbon atoms. Consequently, the O:C ratio is 4/29 = 0.138.

We next determine the expected amount of O if the organic component was purely PIM-1 based on the stoichiometric ratio determined above. This is done by substituting in the expected stoichiometric ratio (0.138) and the at.% C measured in the survey scan (45.4 at%) into the following ratio equation: For PIM-1 the at. % O attributed to the polymer can be determined using the at. % C and the O:C ratio.

$$\text{O:C ratio} = \frac{(at.\ \%O)_{polymer}}{(at.\ \%C)_{polymer}} = \frac{(at.\ \%O)_{polymer}}{45.4\ at\%} = 0.138$$

Thus, the expected amount of oxygen in the organic fraction of the material if it were purely PIM-1 would be 45.4 at% O * 0.138 = 6.2 at% O.

Given this value (6.2 at% O) is smaller than the value measured experimentally above (8 at% O), we attribute the excess oxygen to adsorbed water ligands.

Determining the H_2O:Al Ratio:

We now technically use the O:C ratio to determine the amount of O that can be attributed to water via:

$$\frac{[O\ at\%]_{PIM1} + [O\ at\%]_{H_2O}}{[C\ at\%]_{measured}} = \frac{[O\ at\%]_{measured}}{[C\ at\%]_{measured}} \text{ or}$$

$$\frac{6.2\ at\%\ O + [O\ at\%]_{H_2O}}{45.4\ at\%\ C} = \frac{8\ at\%\ O}{45.4\ at\%\ C},$$

which simply reduces to the difference between the two O at% values calculated in the prior two sections, giving at O at% for water of 1.8 at% O. We then determine a H_2O : Al ratio by:

$$\frac{[O\ at\%]_{H_2O}}{[Al\ at\%]_{measured}} = \frac{1.8\ at\%}{13\ at\%} = 0.138$$

Thus, for the as-synthesized hybrid material, we calculate a H_2O : Al ratio of 0.138.

X-ray Near Edge and Extended Edge Supporting Information

Below is some additional information surrounding XANES & EXAFS.

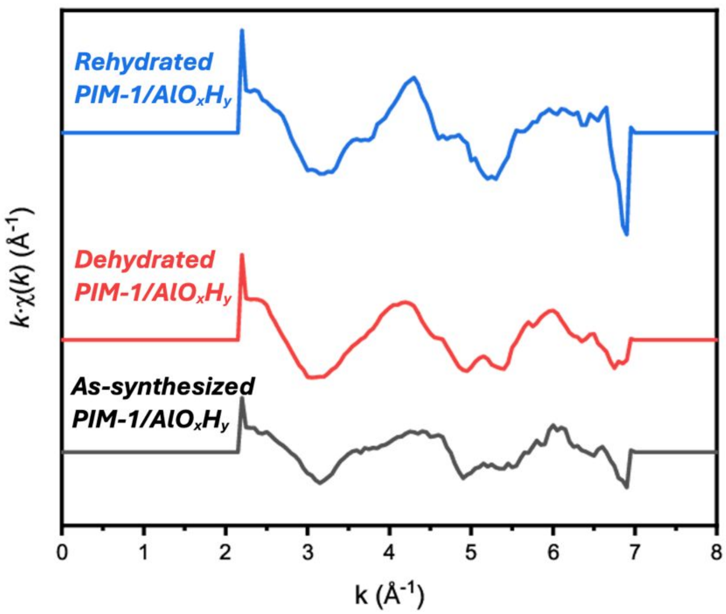

Figure S1: K-space EXAFS spectra for as synthesized PIM-1/AlO_xH_y hybrid membranes, dehydrated PIM-1/AlO_xH_y hybrid membranes, and rehydrated PIM-1/AlO_xH_y hybrid membranes. FT-EXAFS data was generated using a k-range of Δk=2.3-6.5 $Å^{-1}$
